# Research progress on the relationship between autophagy and chronic complications of diabetes

**DOI:** 10.3389/fphys.2022.956344

**Published:** 2022-08-08

**Authors:** Xia Ge, Ling Wang, Aihua Fei, Shandong Ye, Qingping Zhang

**Affiliations:** ^1^ Department of Endocrinology, The Second Affiliated Hospital of Anhui University of Chinese Medicine, Hefei, China; ^2^ Graduate School of Anhui University of Chinese Medicine, Hefei, China; ^3^ Department of Endocrinology, The First Affiliated Hospital of University of Science and Technology of China, Hefei, China; ^4^ College of Acupuncture-Moxibustion and Tuina, Anhui University of Chinese Medicine, Hefei, China

**Keywords:** autophagy, chronic complications, diabetes drug, molecular mechanism, regulation

## Abstract

Diabetes is a common metabolic disease whose hyperglycemic state can induce diverse complications and even threaten human health and life security. Currently, the treatment of diabetes is restricted to drugs that regulate blood glucose and have certain accompanying side effects. Autophagy, a research hotspot, has been proven to be involved in the occurrence and progression of the chronic complications of diabetes. Autophagy, as an essential organismal defense mechanism, refers to the wrapping of cytoplasmic proteins, broken organelles or pathogens by vesicles, which are then degraded by lysosomes to maintain the stability of the intracellular environment. Here, we review the relevant aspects of autophagy and the molecular mechanisms of autophagy in diabetic chronic complications, and further analyze the impact of improving autophagy on diabetic chronic complications, which will contribute to a new direction for further prevention and treatment of diabetic chronic complications.

## Autophagy

**FIGURE 1:**
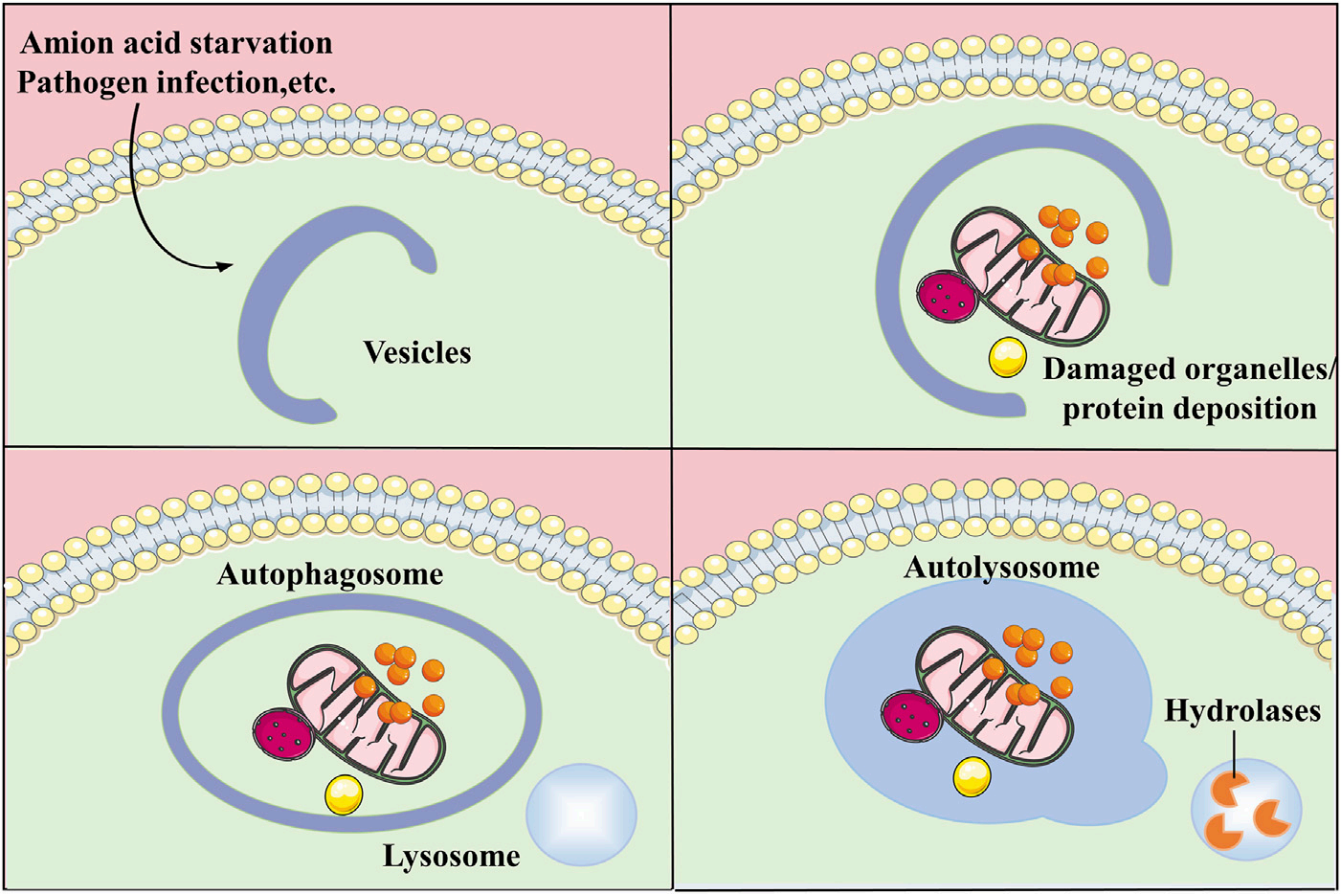
General process of autophagy. Autophagy is a process that engulfs its own cytoplasmic proteins or organelles and encapsulates them into vesicles and fuses with lysosomes to form autolysosomes that degrade their encapsulated contents, realizing the cell’s own metabolic needs and the renewal of certain organelles.

Autophagy is an essential catabolic process in which broken organelles and proteins in cells are wrapped by a double layer of vesicles to form autophagosomes, which then combine with lysosomes to constitute autolysosomes and are finally degraded by a variety of enzymes in the autolysosomes (Kim and Lee, 2014). In addition to the macroautophagy described above, autophagy also includes microautophagy and chaperone-mediated autophagy (CMA). Autophagy is essential for the metabolism of substances and the maintenance of energy homeostasis by degrading biological macromolecules into simple molecules which can re-enter the cycle for the synthesis of other substances. In addition, autophagy degrades lipids to produce free fatty acids which can be oxidised by mitochondria to produce energy (Yang et al., 2019). Autophagy not only facilitates organelle renewal and cellular metabolism by degrading damaged organelles and proteins, but also, is activated in response to adverse extracellular stimuli and assists cells to resist stress. Autophagy is associated with apoptosis and plays a protective function in regulating the cell cycle and maintaining genomic stability (Wu et al., 2011; Yoo and Jung, 2018). The process of autophagy is mediated by multiple pathways and involves a variety of organelles such as mitochondria, endoplasmic reticulum (ER), ribosomes, peroxisomes, and lysosomes. With further studies on autophagy, it has been proved that autophagy can be not only non-selective but also selective. The selective degradation of autophagy can stabilize the cellular state and prevent the occurrence of diseases (Levine and Kroemer, 2019). The disruption of autophagy has an impact on the development of several diseases such as cancer (Ferro et al., 2020), diabetes (Muralidharan and Linnemann, 2021), cardiovascular diseases (Bravo-San Pedro et al., 2017), neurological related diseases (Bingol, 2018) and inflammatory diseases (Yao et al., 2021) (Figure 1).

Autophagy, a cellular degradation pathway, has been proposed for decades. Another cellular degradation pathway, the ubiquitin-proteasome system, is interconnected and even has the same molecules as autophagy (Pohl and Dikic, 2019). The multiple molecular mechanisms of autophagy respond correctly to different cellular states and the energy generated by this process can be utilized by the cells. The dysregulation of autophagy in diabetes can be a potential therapeutic target (Dikic and Elazar, 2018). Autophagy is involved in the regulation of pancreatic β cells, protecting insulin target tissues, and its abnormal regulation may adversely affect the chronic complications of diabetes (Barlow and Thomas, 2015). Hyperglycemia is a characteristic feature of diabetes, and in this state, aberrant regulation of autophagy occurs (Barutta et al., 2022). Abnormalities in autophagy can contribute to various organelle dysfunctions and complications. In this paper, we summarize the molecular mechanisms of autophagy regulation and the latest advances in autophagy in chronic complications of diabetes to offer a reference for its prevention and treatment in the future.

## Classification of autophagy

In mammalian cells, autophagy occurs within the lysosome and can be divided into three types according to the route of transport of substances to the lysosome: microautophagy, macroautophagy and CMA (Ravanan et al., 2017). Microautophagy functions in cellular metabolism, organelle renewal and biosynthesis, and is a type of autophagy in which lysosomes directly wrap around the cytoplasm for degradation without the involvement of other membranes (Li et al., 2012; Schuck, 2020). Studies have demonstrated that microautophagy is also present in plant cells and can degrade organelles such as broken chloroplasts to maintain normal plant cell function (Sieńko et al., 2020). Macroautophagy is the most studied form of autophagy and is the main form of autophagy presented in this paper. Macroautophagy is a process in which proteins and other substances in the cytoplasm are wrapped by two membrane structures to form autophagosomes, which bind to lysosomes to form autolysosomes that degrade the wrapped substances (Feng et al., 2014). The molecular mechanism of autophagy is complex and is associated with cellular states and the development of many diseases. It operates in the immune system by affecting antigen processing and antigen presentation processes (Münz, 2021). Autophagy facilitates the renewal of destroyed organelles and other cytoprotective functions associated with human aging. The protective effects of autophagy and autophagy on cells are complicated with ageing (Nieto-Torres and Hansen, 2021). Recent studies have shown that the regulation of proteins by neuronal autophagy is in association with the aging of neuronal cells (Kallergi and Nikoletopoulou, 2021). CMA is a process of selective autophagy for specific proteins which contain a specific targeting motif in its amino acid sequence that can be combined with the heat shock cognate 70 (HSC70) protein and finally transported to the lysosome for degradation. (Kaushik and Cuervo, 2018). CMA can be a potential target to regulate the function of hematopoietic stem cells and is integral to the proper functioning of part of the normal function of hematopoietic stem cells (Dong et al., 2021). Endosomal microautophagy, a selective autophagy that occurs through endosomal autophagy, and HSC70 chaperones specifically bind to the KFERQ-like motif partially similar to CMA (Tekirdag and Cuervo, 2018; Krause and Cuervo, 2021).

## Regulatory factors of autophagy and diabetes

Autophagy-associated genes (ATGs) are central to the molecular machinery of autophagy and are involved in the encoding of key proteins that play an active role in signal transduction pathways. Furthermore, ATG, whose aberrant expression can induce disease, play important functions in different autophagic processes such as cytoplasmic encapsulation, autophagic vesicle formation and lysosomal fusion (Levine and Kroemer, 2019; Mizushima and Levine, 2020). ATG5 and LC3 are key genes of autophagy and their levels are obviously lower in diabetic nephropathy (DN) model mice than those in normal group, which affects autophagy (Yassin et al., 2021). Autophagy is an essential regulatory mechanism in diabetes, where the serum levels of the ATG protein Beclin-1 are inversely correlated with carotid intima-media thickness in patients with type 2 diabetes (T2D) (Naguib et al., 2021). The hyperglycemic state of diabetic rats increases the accumulation and expression of Beclin-1 simultaneously promoting retinal Müller cell death in rats (Lopes de Faria et al., 2016). Beclin-1 and Bcl-2 are interrelated and their binding downregulates Beclin-1-mediated autophagy in diabetic nephropathy (Xu and Qin, 2019; Liu et al., 2022). TFEB, a regulatory factor, is associated with lysosomal biosynthesis. The accumulation of phosphorylated TFEB in cells reduces TFEB activity which in turn impacts autophagy. Mechanistic target of rapamycin (mTOR) regulates autophagy and lysosomal biosynthesis through phosphorylation of TFEB (Sha et al., 2017). TFEB promotes macrophage M2 polarization for restoring the cytoprotective function of autophagy in mesenchymal stem cells of DN mice (Yuan et al., 2020). Studies have shown that TFEB and glucose metabolism are closely related, with TFEB knockdown reducing glucose uptake in endothelial cells by about half compared to controls, while increasing TFEB expression increases glucose uptake exponentially (Sun et al., 2021).

Phosphorylated Akt can inhibit autophagy by activating mTOR complex 1 (mTORC1) and inhibiting FOXO1, playing an important role between insulin signaling and autophagy regulation. In animal experiments using mice with adipocyte-specific innate knockout of Atg7 and mice with adipocyte-specific knockout of Atg1611 or Atg3, respectively, it was concluded that innate inhibition of autophagy affects adipocyte differentiation, promotes adipocyte browning and increases insulin sensitivity, but selective inhibition of autophagy in mature adipocytes leads to insulin resistance (Frendo-Cumbo et al., 2021). Studies on excessive autophagy have found that excessive activation of autophagy inhibits insulin secretion by *β* cells on the one hand, but increases insulin sensitivity by reducing ER stress in insulin-responsive cells on the other (Yamamoto et al., 2018).

## Autophagy and the chronic complications of diabetes

**FIGURE 2:**
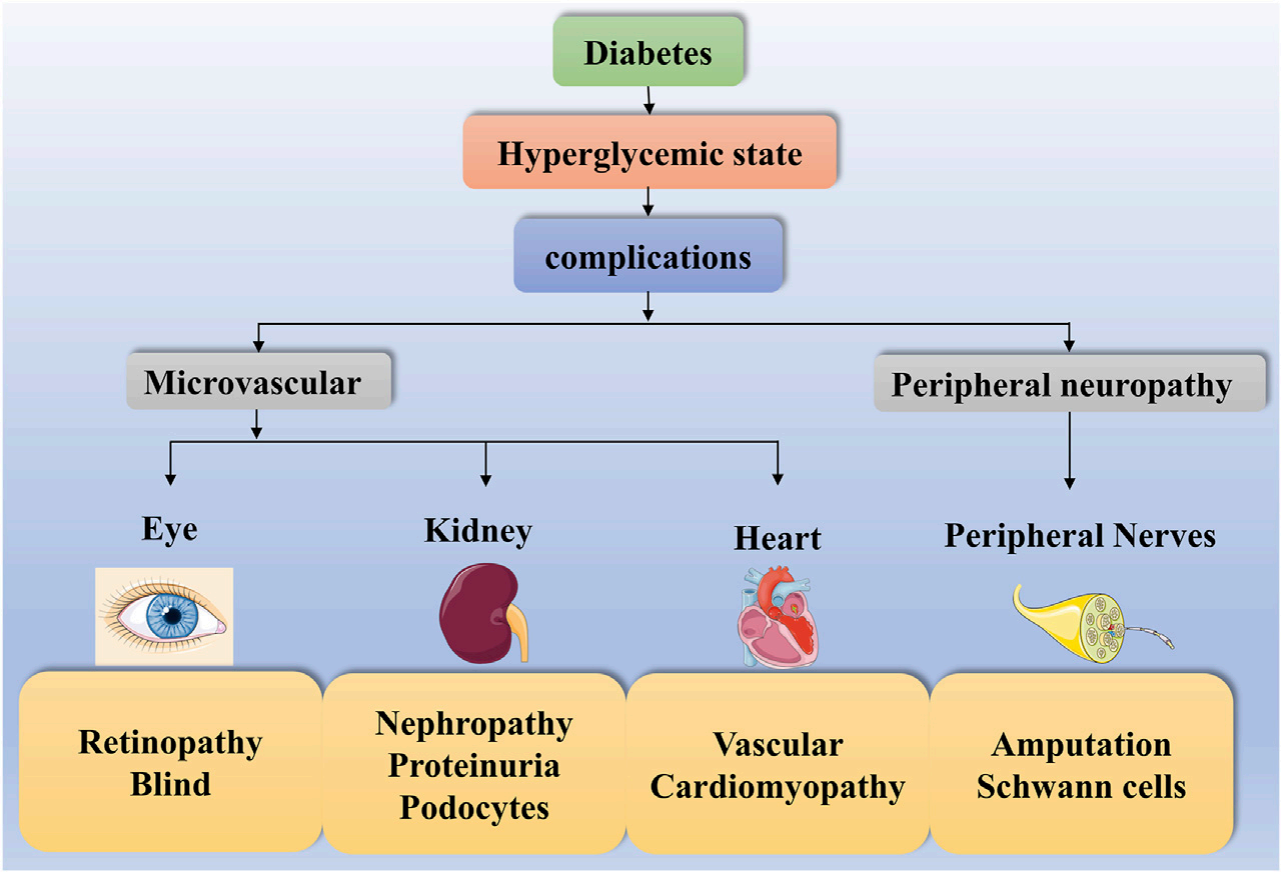
Diabetes complications. The high-glucose state of diabetes can contribute to diverse complications such as diabetic microangiopathy and peripheral neuropathy. Some of these diabetic microangiopathies involve retinopathy, nephropathy and heart disease, such as Retinopathy, Blind, Nephropathy, Proteinuria, Podocytes, Vascular and Cardiomyopathy. In contrast, peripheral neuropathy presents with Amputation and Schwann cells.

Diabetes is a common metabolic disease with hyperglycemia. According to statistics, the global incidence of diabetes has been increasing since 1980 and has now doubled (NCD-RisC, 2016). Hyperglycemia triggers various metabolic signaling pathways leading to autophagy, inflammation and even cell death. This disruption of cellular metabolism contributes to diverse diabetic complications. Complications such as diabetic retinopathy (DR), DN, diabetic heart disease and diabetic peripheral neuropathy (DPN) are highly prevalent in both type 1 and type 2 diabetics, increasing the risk of diabetes and even leading to death (Forbes and Cooper, 2013; Cole and Florez, 2020) (Figure 2).

In addition to the major complications of diabetes, Alzheimer’s disease (AD) is closely associated with T2D, which is referred to by some as type 3 diabetes. Insulin resistance and insulin signalling dysfunction in T2D are important risk factors for dementia and AD. Peripheral and central insulin resistance leads to reduced insulin signalling, increased Aβ toxicity, oxidative stress and even the development of neurodegeneration. Even in non-diabetic people, insulin resistance increases the risk of dementia and AD (Nguyen et al., 2020).

### Diabetic retinopathy

DR is a common diabetic complication associated with microangiopathy, which can result in further health impairment in diabetic patients with other vascular diseases (Cheung et al., 2010). The later stages of DR can contribute to blindness. It is beneficial to be acquainted with the mechanisms involved in DR to prevent and treat the disease in order to reduce the rate of blindness (Lin et al., 2021). Factors associated with hyperglycaemia, oxidative stress, hypoxia and ER stress in DR are closely associated with autophagy (Gong et al., 2021).

Abnormal autophagy due to high glucose promotes the progression of DR. High glucose facilitates ROS production by impacting mitochondrial function, and the resulting high ROS levels and damaged mitochondria can induce oxidative stress. Oxidative stress is one of the molecular mechanisms of autophagy, which can activate autophagy in DR. Appropriate stress can be used to improve cellular status by regulating autophagy, whereas excessive stress has the opposite effect in facilitating disease progression (Fu et al., 2016; Rosa et al., 2016; Dehdashtian et al., 2018). High glucose status promotes autophagosome production and regulates autophagy through ROS-mediated ER stress signaling (Yao et al., 2014). In DR, high mobility group box 1 (HMGB1) is involved in lysosomal membrane disruption through a cathepsin B-dependent pathway, thereby inhibiting autophagy. Inhibition of HMGB1 expression in retinal pigment epithelial cells under high glucose conditions resulted in downregulation of cathepsin B expression, thereby restoring the dysfunction of the autophagic lysosomal pathway (Feng et al., 2021). The high glucose state can result in elevated levels of vascular endothelial growth factor, thereby promoting neointima generation in DR. Autophagy can reduce vascular endothelial growth factor expression acting as a cytoprotective agent (Lopes de Faria et al., 2016).

### Diabetic nephropathy

DN, a chronic complication of diabetes mellitus, is impacted by glycemic, lipid, lifestyle and genetic factors, for which proteinuria, neutrophil gelatinase-associated adiponectin, cystatin C and plasma growth differentiation factor 15 are several representative markers (Papadopoulou-Marketou et al., 2017). The hyperglycemic state of diabetes affects cellular metabolism, which leads to the development of pathological symptoms such as advanced glycosylation end products and glomerular hypertension, facilitating the DN along with other systemic diseases to further develop end-stage renal disease (Wada and Makino, 2013; Xiong and Zhou, 2019). Aberrant regulation of autophagy is associated with these multiple adverse progressions of diabetic nephropathy (Yang D et al., 2018).

Abnormal autophagy caused by high glucose promotes DN progression. The homeostasis of podocytes is related to autophagy in which the removal of Atg5 in podocytes would impact the cellular state of podocytes contributing to the inability of cells to resist stimuli induced by high glucose, a change which also exists in endothelial cells. The high glucose state in diabetes increases the levels of the autophagy marker LC3B II in podocytes with the consequent activation of autophagy which is uncertain whether it protects the kidney (Lenoir et al., 2015; Liu et al., 2018). However, high glucose could also contribute to resulting autophagy defects and proteinuria production (Tagawa et al., 2016). Mesenchymal stem cell-derived exosomes can modulate the levels of autophagy-related markers in diabetic rats and reduce renal impairment by activating autophagy for the treatment of DN (Ebrahim et al., 2018). miR-486 in disaccharide stem cell-derived exosomes can inhibit mTOR activation by decreasing Smad1 expression, thereby increasing autophagy and protecting podocytes (Jin et al., 2019). In DN, mTOR inhibits the formation of autophagosomes in podocytes by promoting advanced glycation end-products (AGEs) to suppress nuclear translocation and activity of TFEB, achieving a regulatory role in autophagy (Zhao et al., 2018). Recent studies have identified a possible mechanism of autophagy dysregulation in DN, where SMAD3 binds to the 3′-UTR of TFEB and represses its transcription, and a decrease in TFEB leads to a decrease in lysosome synthesis. Conversely, when SMAD3 is inhibited, the inhibition of lysosomal biosynthesis is reduced, thereby regulating the level of autophagy in DN (Yang et al., 2021).

### Diabetic heart disease

Lesions of blood vessels in diabetic patients can induce heart-related problems such as heart failure through oxidative stress and inflammatory stimulation, which greatly increase mortality in diabetic patients. Vascular disease is even the most common cause of death in patients with T2D (Blendea et al., 2003; Ritchie and Abel, 2020). The symptoms of heart disease in diabetic patients are not obvious in the early stages, increasing the difficulty of treatment when detected later. Therefore, it is significant to explore the mechanisms of diabetic heart disease for early detection of the disease (Rawal et al., 2014). Autophagy plays an essential role in diabetic cardiomyopathy, where disturbances in cardiac metabolism and inhibition of lysosomal degradation contribute to the disruption of autophagy (Kanamori et al., 2021).

The high levels of oxidative stress generated in the diabetic heart can induce mitochondrial dysfunction, while mitochondrial damage and autophagy inhibition can in turn exacerbate diabetic heart disease (Tong et al., 2019). Autophagy protects cardiomyocytes and ameliorates myocardial injury by removing abnormal mitochondria (Kubli and Gustafsson, 2015). miRNA, which is associated with protein expression, has a mechanism of effect associated with autophagy and is shown to act in cardiac disease (Sermersheim et al., 2017). In diabetic mice, lncRNA AK139328 affected myocardial ischemia-reperfusion injury by regulating the level of autophagy. lncRNA AK139328 exacerbated myocardial ischemia-reperfusion injury in diabetic mice by promoting autophagy *via* suppressing the expression level of miR-204-3p (Yu et al., 2018). lncRNA DCRF, a similar effect as hyperglycemia, facilitates the expression of DCRF thereby promoting autophagy in cardiac cells and exacerbating myocardial injury. In addition, DCRF can compete with PCDH17 as a target of miR-551B-5p (Feng et al., 2019).

### Diabetic peripheral neuropathy

DPN, a chronic complication of diabetes mellitus, usually refers to lower extremity neuropathy. It is the leading cause of amputation associated with diabetes and has a worse prognosis (Selvarajah et al., 2019). Autophagy plays an active role in neurodegenerative diseases and neurological tissue damage. In the peripheral nerves of hypoglycemic diabetic rats, autophagy is observed both in the early stages of axonal degeneration and in the regeneration process, especially in the regenerating axons (Mohseni, 2011).

Abnormal autophagy induced by high glucose promotes the progression of DPN. Enhanced miR-30d-5p in trigeminal sensory neurons of diabetic mice inhibited the levels of autophagy markers LC3II and Beclin-1. In the high glucose state, upregulation of X-inactive specific transcript of lncRNA induced autophagy which was inhibited by MiR-30d-5p thereby dealing with oxidative stress in Schwann cells and attenuating DPN (Liu et al., 2021). In DPN, HDAC1 inhibited the expression of autophagy markers LC3-II/LC3-I and P62 when high glucose treated Schwann cell 96 thereby suppressing autophagy (Du et al., 2019). Thioredoxin-interacting protein was associated with neurotransmission and other dysfunctions in Schwann cells. Thioredoxin-interacting protein expression was significantly increased in Schwann cells under the effect of high glucose and inhibited the expression of autophagic markers, which aggravated the process of DPN (Zhang X et al., 2021).

### Classical mechanisms associated with the development of chronic complications of diabetes

AGEs associated with hyperglycaemia play an important role in the development of diabetic complications, and in DN the accumulation of AGEs and their receptors can further impair renal function (Sanajou et al., 2018). In addition, AGEs can stimulate the expression of autophagy-related proteins in diabetic rat dermal fibroblasts and human fibroblasts and induce autophagy, providing some support for the demonstration that AGEs contribute to the development of diabetic ulcers. (Sun et al., 2016). Beclin-1 and Bcl-2 are targets of O-Glcnacylation. Increased hexamine biosynthesis pathway fluxes and elevated protein O-Glcnacylation are associated with impaired autophagy signaling in db/db diabetic mouse cardiomyocytes (Marsh et al., 2013). Under the same culture conditions, erythrocytes from patients with type 1 diabetes accumulated more sorbitol than normal erythrocytes over the same time period. The majority of diabetic patients have an increased accumulation of sorbitol, a phenomenon associated with the development of diabetes-related complications (Malone et al., 1984). The hyperglycaemic state of diabetes leads to increased diacylglycerol (DAG) levels and protein kinase C (PKC) activation. The DAG-PKC pathway can affect cardiovascular function and accelerate the progression of diabetic complications by regulating endothelial permeability, vasoconstriction and cytokine activation in various ways (Das Evcimen and King, 2007). Some of these glycaemia-dependent pathways for the development of chronic complications of diabetes have mechanisms of action related to the regulation of autophagy. For example, PKCα negatively regulates autophagy in diabetic pregnancy, and removal of PKCα eliminates maternal diabetes-induced neuroepithelial apoptosis and neural tube defect formation (Wang et al., 2017).

## Molecular mechanisms of autophagy associated with chronic complications of diabetes

**FIGURE 3:**
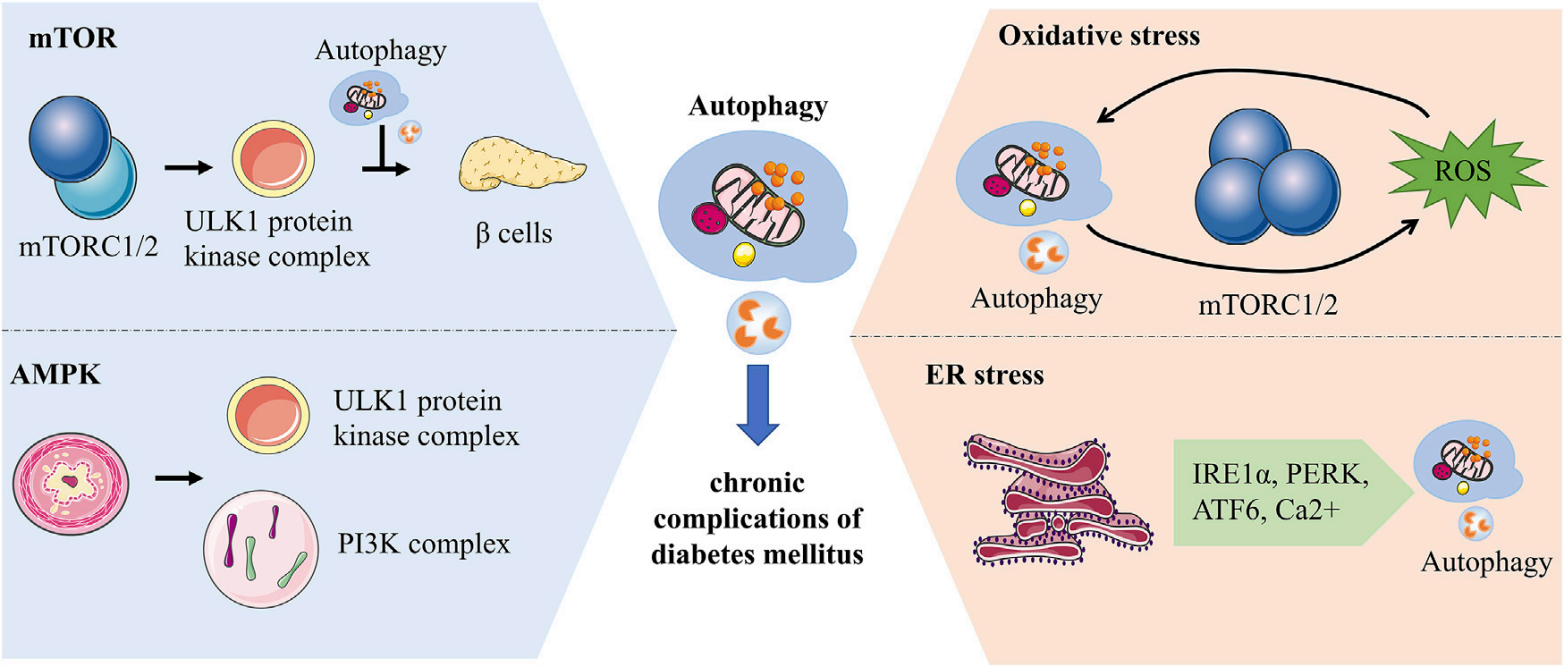
Molecular mechanisms of autophagy associated with chronic complications of diabetes. mTORC1 regulates the initiation of autophagy by inhibiting the phosphorylation of the ULK1 complex and has a bidirectional effect on pancreatic β-cells. AMPK can impact autophagy by activating phosphorylation of ULK1 or regulating the PI3K complex. Autophagy and ROS can be mediated by each other. ROS activates autophagy by increasing beclin-1 expression and over-suppressing mTORC1, while autophagy can maintain ROS levels by clearing mitochondria. ER is an essential site of protein folding, while ER stress-mediated autophagy is associated with IRE1α, PERK, ATF6 and Ca^2+^ signaling pathways.

Recently, researches at the molecular level of autophagy in yeast and eukaryotes have been considerably explored. In chronic complications of diabetes, autophagy is mediated by diverse pathways such as the mTOR, AMP-activated protein kinase (AMPK) and different stresses. Autophagy is dramatically increased in response to cellular stresses, such as oxidative stress and ER stress. It provides assistance to cells in handling stress and restoring stability to the internal environment by removing faulty proteins as well as harmful substances (Figure 3).

### Mechanistic target of rapamycin

mTORC1 and mTORC2 are key components of mTOR which activate different downstream signals to mediate autophagy (Kim and Guan, 2015). ULK proteins, homologs of yeast ATG1, are involved in autophagosome formation and influence the recruitment of autophagy-associated proteins (Hara et al., 2008; Mizushima, 2010). The ULK1 protein kinase complex can receive signals to initiate autophagy, while mTORC1 inhibits the initiation of autophagy by inhibiting the phosphorylation of the ULK1 complex thereby regulating autophagy (Al-Bari and Xu, 2020). Hyperactive mTORC1 in the pancreas has a bidirectional regulatory effect on islet *β* cells. Overactivation of mTORC1 initially promotes increased *β* cell mass, increased insulin levels, and improved glucose tolerance, but over time this promoting effect becomes inhibitory and *β* cell function declines more rapidly (Saxton and Sabatini, 2017). mTOR inhibitors act on the mTOR signaling pathway leading to elevated blood glucose by affecting insulin secretion. It is effective in combination with metformin to prevent diabetes caused by its use alone (Vergès and Cariou, 2015; Kezic et al., 2018).

### AMP-activated protein kinase

AMPK, an energy-related enzyme, functions in autophagy by regulating the phosphorylation of autophagy-associated proteins. AMPK maintains energy homeostasis through enzymatic regulation of energy regulatory pathways (Tamargo-Gómez and Mariño, 2018). In contrast to mTOR, the AMPK pathway promotes autophagy. In hypoglycemic states, AMPK activates Ulk1 to initiate autophagy through phosphorylation of Ser 317 and Ser 777. mTOR can influence the relationship between Ulk1 and AMPK to negatively regulate autophagy (Kim et al., 2011). Co-activator-associated arginine methyltransferase 1 is an essential part of autophagy whose levels are regulated by SKP2. In the presence of nutrient deficiency, AMPK-dependent phosphorylation of FOXO3a inhibits SKP2 transcription thereby increasing the level of Co-activator-associated arginine methyltransferase 1, a novel signaling axis (Shin et al., 2016). PI3K class III protein complexes are important for autophagosome formation, while VPS34 is a catalytic subunit. AMPK can influence autophagy by regulating the PI3K complex to affect autophagy. AMPK phosphorylates Beclin1 at Thr388 and promotes Beclin1 binding to VPS34 and ATG14 (Kim and Guan, 2013). In the absence of nutrients, AMPK phosphorylates Ser-91 and Ser-94 of Beclin1 and promotes autophagosome formation (Kim et al., 2013). AMPK regulates autophagy, apoptosis, mitochondrial homeostasis and other cellular metabolic processes (Herzig and Shaw, 2018). AMPK can improve diabetic cardiomyopathy by promoting fatty acid *β*-oxidation *via* the ACC-CPT-1 pathway (Yang H et al., 2018). AMPK regulates lipid metabolism in diabetes by inhibiting the degradation of Insig-1 and causing a reduction in the expression of adipogenesis-related genes, which can be reversed by AMPK activators. A769662, an AMPK activator, is a potential drug for the treatment of chronic complications of diabetes (Han et al., 2019; Madhavi et al., 2019). Metformin, another AMPK agonist, increases autophagic flux in cardiomyocytes to reduce the burden of cardiomyopathy in patients with type 2 diabetes (Packer, 2020).

### Oxidative stress

Autophagy is sensitive to REDOX signaling. Reactive oxygen species (ROS), a signal that maintains autophagy, which functions in the cell depending on the concentration. Low concentrations of ROS can act as REDOX signals, while high concentrations of ROS can injure cells (Yun et al., 2020). High levels of ROS produced by damaged mitochondria can react on mitochondria to clean up damaged mitochondria and maintain cellular homeostasis by activating autophagy (Filomeni et al., 2015). Metabolic inflammation in diabetes is associated with autophagy induced by oxidative stress. Blood glucose fluctuations in diabetic patients increase oxidative stress and chronic inflammation in endothelial cells (Chang et al., 2012; Muriach et al., 2014). Autophagy and ROS can mediate each other. ROS activates autophagy by increasing beclin-1 expression and over-suppressing mTORC1, while autophagy can maintain ROS levels by clearing mitochondria (Alexander et al., 2010; Lavallard et al., 2012). The mutual regulation of autophagy and ROS is multi-pathway, in which ROS can activate autophagy through different pathways at the transcriptional and translational levels (Li et al., 2015).

### Endoplasmic reticulum stress

ER senses stress in the internal and external environment as well as participates in the synthesis and transport of intracellular substances, a feature associated with autophagic mechanisms in diabetes (Su et al., 2013). ER stress can induce interrelated autophagy and endoplasmic reticulum phagocytosis. ER stress-mediated autophagy is associated with the IRE1α, PERK, ATF6, and Ca^2+^ signaling pathways (Song et al., 2018). ER is an essential site of protein folding, where nutritional stressors such as inadequate nutrition, Ca^2+^ metabolic imbalance, and oxidative stress may affect its cellular function, leading to the accumulation of unprocessed proteins and thus activating the unfolded protein response (UPR) (Bhardwaj et al., 2020). ER stress protects cells by activating autophagy, which removes unfolded proteins and damaged ER. At the same time, UPR activates transcription factors to regulate the expression of autophagy-related proteins (Deegan et al., 2013). UPR helps ER repair mishandled proteins by activating PERK, IRE1, and ATF6. When the repair limit of UPR is exceeded, the damaged ER is degraded by autophagy and the degraded ER fragments can be recycled (Qi and Chen, 2019). It has been reported that ER is associated with the size of autophagosome formation and ATG2 transfers lipid molecules from ER and participates in autophagosome formation (Yamamoto and Noda, 2020). The ER stress pathway may be associated with diabetes-related cognitive decline, but in streptozotocin-induced diabetic mice, ER stress-mediated autophagy induced by the JNK pathway may protect neurons (Kong et al., 2018).

## Effect of improved autophagy on chronic complications of diabetes

With the epidemic of diabetes, a large number of novel hypoglycemic agents have been developed, some of which can regulate autophagy through varying signaling pathways and are useful in the treatment of chronic complications of diabetes (Zhang et al., 2017).

### Improve autophagy to treat diabetes

Dispo85E has been shown to be a potential drug for the treatment of diabetic vascular complications, and the mechanism by which it acts is associated with autophagy. In a diabetic mouse model, Dispo85E facilitates the expression of hepatocyte growth factor in non-platelet cells, thereby promoting the degradation of AGEs by hepatocyte growth factor-induced autophagy, which has a therapeutic effect on diabetic complications (Peng et al., 2011). Pioglitazone, a receptor gamma agonist, ameliorates the consequences of cellular damage caused by the accumulation of AGEs in diabetes by promoting autophagy (Xu et al., 2020). In T2D, AMPK and SIRT1 signaling is inhibited, whereas metformin and SGLT-2 inhibitors can activate both signals to facilitate autophagy, respectively (Packer, 2020). The therapeutic effect of metformin in autophagy regulation in diabetes is associated with TFEB, which regulates metformin-induced autophagy (Zhang D et al., 2021). MSL, an autophagy enhancer, activates calcineurin and increases the conversion of LC3-I to LC3-II. It regulates autophagy by inducing dephosphorylation/nuclear translocation of TFEB to achieve therapeutic effects in diabetic mice (Lim et al., 2018).

In T2D mice, the SGLT-2 inhibitor empagliflozin and the DPP-4 inhibitor linagliptin used alone or in combination enhance the levels of markers associated with glomerular autophagy and contribute to the recovery of autophagy, thus reducing the damage to the kidney in diabetes (Korbut et al., 2020). Glucagon-like peptide 1 (GLP-1) receptor agonist-Liraglutide may promote autophagy through regulating the AMPK/mTOR pathway to exert renoprotective effects in a rat remnant kidney model of chronic renal failure (Xue et al., 2019). In addition, the GLP-1 receptor agonist exendin-4 prevents *β* cell loss in type 2 diabetes by restoring lysosomal function and autophagic flux and facilitates the maintenance of glucose homeostasis. Exendin-4 also alleviates tacrolimus-induced hyperglycaemia, oxidative stress and other states of autophagic overload by activating autophagosomal clearance (Lim et al., 2016; Zummo et al., 2017; Arden, 2018). For cardiomyopathy, GLP-1RAs improve mitochondrial function *via* regulating autophagy and inflammatory signaling, as well as not only mediate the inhibition of myocardial apoptosis, but also improve cardiac energy metabolism (Ma et al., 2021). Melatonin, a neurohormone with diverse functions, can ameliorate the impact of DR by activating autophagy and regulating cellular status. Melatonin can maintain cellular stability by ameliorating hyperglycemia-induced cellular stress and disruption of the blood-retinal intra-retinal barrier (Yan et al., 2021).

### Improve autophagy to treat cognitive impairment

Apoptosis regulator (TP53-inducible glycolysis and apoptosis regulator, TIGAR), an inhibitor of glycolysis, its expression level is decreased in high glucose state. High expression of TIGAR mitigates autophagy damage induced by high glucose and positively regulates autophagy. TIGAR protects neuronal cells through autophagy in diabetic neuropathy and attenuates cognitive impairment (Zhou et al., 2019). Increased expression of HMGB1 protein and defective autophagy in type 2 diabetic mice during intermittent hypoxia exacerbate cognitive impairment (Guo et al., 2019). Liraglutide protects neurons and improves diabetes-induced cognitive impairment by ameliorating hippocampal neuronal and synaptic damage (Yan et al., 2019). Metformin or pioglitazone can improve cognitive function induced by diabetic vascular injury by modulating neuronal autophagic flux (Fakih et al., 2020). Rosiglitazone increases the survival rate of beta cells in diabetic patients by promoting AMPK phosphorylation to activate autophagy (Wu et al., 2013).

In a study of newly diagnosed T2D patients in Korea, there were 1675 AD cases with 8375 control cases. The study suggests that metformin use is associated with an increased incidence of AD in patients with type 2 diabetes and that the duration of diabetes increases the risk of AD (Ha et al., 2021). Interestingly, another study has shown that the use of metformin is beneficial in improving the cognitive status of people with T2D and that adherence to Mediterranean diet may be more effective than metformin (Soldevila-Domenech et al., 2021). In the APP/PS1 mouse model of AD, metformin activates CMA *via* the TAK1-IKKα/β-Hsc70 signalling pathway and effectively reduces the accumulated brain Aβ plaques, providing a therapeutic effect in AD (Xu et al., 2021). Based on the above studies, the alleviating effect of metformin on cognitive impairment in diabetic patients may be related to its modulation of autophagy. In a clinical trial of metformin in non-diabetic patients, venous blood and stool were collected and evaluated in subjects taking metformin within 90 days and after 30 days, and the results showed additional anti-inflammatory, anti-aging and anti-thrombotic properties of metformin (Zuckerbraun, 2018). In addition, in a trial on the effect of metformin on surrogate markers of cellular senescence and autophagy in adults with prediabetes, a 12-weeks trial of metformin versus CaCO3 (placebo) was conducted and the results so far are that LC3 levels in leukocytes were increased in the metformin group, indicating enhanced autophagic activity (Burge, 2017).

## Conclusion

Autophagy is an essential metabolic pathway *in vivo* with a vital role in the chronic complications of diabetes. However, its specific mechanisms have not been fully explored, with relevant theories requiring further research. Although some studies have shown that complete remission is possible in T2D patients treated with intensive lifestyle changes, this study has many limitations and further improvements are needed in diabetes research (Kelly et al., 2020). For diabetes, first of all, we should strictly control blood sugar so that complications can be prevented. Then we have to do more research on diabetes in the field of metabolism to figure out the metabolic mechanisms and vital markers of chronic complications of diabetes.

Overall, therapeutic approaches to diabetes targeting autophagy need to be better designed. In this review, we describe the molecular mechanisms associated with autophagy and diabetic complications and summarise the latest autophagy-related drugs to improve diabetes and its complications. These results highlight the role of autophagy in the amelioration of diabetic complications and contribute to the research and application of glucose-lowering drugs related to the molecular mechanisms of autophagy. Currently, common approaches to modulating autophagy in diabetes are the use of autophagy inhibitors or knocking out autophagy-related genes, which have shown good modulation in animal experiments but still lack clinical trials. Therefore, further research is still needed on drugs that modulate autophagy, and modulating autophagy may be a potential research direction for the treatment of chronic complications of diabetes.

## Abbreviations

AD, Alzheimer’s disease; AGEs, Advanced glycation end-products; AMPK, AMP-activated protein kinase; ATGs, Autophagy-associated genes; CMA, Chaperone-mediated autophagy; DAG, diacylglycerol; DN, Diabetic nephropathy; DR, Diabetic retinopathy; DPN, Diabetic peripheral neuropathy; ER, Endoplasmic reticulum; GLP-1, Glucagon-like peptide 1; HMGB1, high mobility group box 1; HSC70, Heat shock cognate 70; mTORC1, mTOR complex 1; mTOR, Mechanistic target of rapamycin; PKC, protein kinase C; ROS, Reactive oxygen species; ROS, Reactive oxygen species; T2D, Type 2 diabetes; UPR, Unfolded protein response.

## References

[B1] Al-Bari M. A. A. Xu P. (2020). Molecular regulation of autophagy machinery by mTOR-dependent and -independent pathways. Ann. N. Y. Acad. Sci. 1467 (1), 3–20. 10.1111/nyas.14305 31985829

[B2] Alexander A. Cai S. L. Kim J. Nanez A. Sahin M. MacLean K. H. (2010). ATM signals to TSC2 in the cytoplasm to regulate mTORC1 in response to ROS. Proc. Natl. Acad. Sci. U. S. A. 107 (9), 4153–4158. 10.1073/pnas.0913860107 20160076 PMC2840158

[B3] Arden C. (2018). A role for Glucagon-Like Peptide-1 in the regulation of β-cell autophagy. Peptides 100, 85–93. 10.1016/j.peptides.2017.12.002 29412836

[B4] Barlow A. D. Thomas D. C. (2015). Autophagy in diabetes: β-Cell dysfunction, insulin resistance, and complications. DNA Cell Biol. 34 (4), 252–260. 10.1089/dna.2014.2755 25665094

[B5] Barutta F. Bellini S. Kimura S. Hase K. Corbetta B. Corbelli A. (2022). Protective effect of the tunneling nanotube-TNFAIP2/M-sec system on podocyte autophagy in diabetic nephropathy. Autophagy, 1–20. 10.1080/15548627.2022.2080382 PMC9851239 35659195

[B6] Bhardwaj M. Leli N. M. Koumenis C. Amaravadi R. K. (2020). Regulation of autophagy by canonical and non-canonical ER stress responses. Semin. Cancer Biol. 66, 116–128. 10.1016/j.semcancer.2019.11.007 31838023 PMC7325862

[B7] Bingol B. (2018). Autophagy and lysosomal pathways in nervous system disorders. Mol. Cell. Neurosci. 91, 167–208. 10.1016/j.mcn.2018.04.009 29729319

[B8] Blendea M. C. McFarlane S. I. Isenovic E. R. Gick G. Sowers J. R. (2003). Heart disease in diabetic patients. Curr. Diab. Rep. 3 (3), 223–229. 10.1007/s11892-003-0068-z 12762970

[B9] Bravo-San Pedro J. M. Kroemer G. Galluzzi L. (2017). Autophagy and mitophagy in cardiovascular disease. Circ. Res. 120 (11), 1812–1824. 10.1161/circresaha.117.311082 28546358

[B10] Burge M. R. (2017). A double-blind, placebo-controlled trial of anti-aging, pro-autophagy effects of metformin in adults with prediabetes. NCT03309007. Albuquerque: University of New Mexico.

[B11] Chang C. M. Hsieh C. J. Huang J. C. Huang I. C. (2012). Acute and chronic fluctuations in blood glucose levels can increase oxidative stress in type 2 diabetes mellitus. Acta Diabetol. 49, S171–S177. 10.1007/s00592-012-0398-x 22547264

[B12] Cheung N. Mitchell P. Wong T. Y. (2010). Diabetic retinopathy. Lancet 376 (9735), 124–136. 10.1016/s0140-6736(09)62124-3 20580421

[B13] Cole J. B. Florez J. C. (2020). Genetics of diabetes mellitus and diabetes complications. Nat. Rev. Nephrol. 16 (7), 377–390. 10.1038/s41581-020-0278-5 32398868 PMC9639302

[B14] Das Evcimen N. King G. L. (2007). The role of protein kinase C activation and the vascular complications of diabetes. Pharmacol. Res. 55 (6), 498–510. 10.1016/j.phrs.2007.04.016 17574431

[B15] Deegan S. Saveljeva S. Gorman A. M. Samali A. (2013). Stress-induced self-cannibalism: on the regulation of autophagy by endoplasmic reticulum stress. Cell. Mol. Life Sci. 70 (14), 2425–2441. 10.1007/s00018-012-1173-4 23052213 PMC11113399

[B16] Dehdashtian E. Mehrzadi S. Yousefi B. Hosseinzadeh A. Reiter R. J. Safa M. (2018). Diabetic retinopathy pathogenesis and the ameliorating effects of melatonin; involvement of autophagy, inflammation and oxidative stress. Life Sci. 193, 20–33. 10.1016/j.lfs.2017.12.001 29203148

[B17] Dikic I. Elazar Z. (2018). Mechanism and medical implications of mammalian autophagy. Nat. Rev. Mol. Cell Biol. 19 (6), 349–364. 10.1038/s41580-018-0003-4 29618831

[B18] Dong S. Wang Q. Kao Y. R. Diaz A. Tasset I. Kaushik S. (2021). Chaperone-mediated autophagy sustains haematopoietic stem-cell function. Nature 591 (7848), 117–123. 10.1038/s41586-020-03129-z 33442062 PMC8428053

[B19] Du W. Wang N. Li F. Jia K. An J. Liu Y. (2019). STAT3 phosphorylation mediates high glucose-impaired cell autophagy in an HDAC1-dependent and -independent manner in Schwann cells of diabetic peripheral neuropathy. Faseb J. 33 (7), 8008–8021. 10.1096/fj.201900127R 30913399

[B20] Ebrahim N. Ahmed I. A. Hussien N. I. Dessouky A. A. Farid A. S. Elshazly A. M. (2018). Mesenchymal stem cell-derived exosomes ameliorated diabetic nephropathy by autophagy induction through the mTOR signaling pathway. Cells 7 (12), E226. 10.3390/cells7120226 30467302 PMC6315695

[B21] Fakih W. Mroueh A. Salah H. Eid A. H. Obeid M. Kobeissy F. (2020). Dysfunctional cerebrovascular tone contributes to cognitive impairment in a non-obese rat model of prediabetic challenge: role of suppression of autophagy and modulation by anti-diabetic drugs. Biochem. Pharmacol. 178, 114041. 10.1016/j.bcp.2020.114041 32439335

[B22] Feng L. Liang L. Zhang S. Yang J. Yue Y. Zhang X. (2021). HMGB1 downregulation in retinal pigment epithelial cells protects against diabetic retinopathy through the autophagy-lysosome pathway. Autophagy 18, 320–339. 10.1080/15548627.2021.1926655 34024230 PMC8942416

[B23] Feng Y. He D. Yao Z. Klionsky D. J. (2014). The machinery of macroautophagy. Cell Res. 24 (1), 24–41. 10.1038/cr.2013.168 24366339 PMC3879710

[B24] Feng Y. Xu W. Zhang W. Wang W. Liu T. Zhou X. (2019). LncRNA DCRF regulates cardiomyocyte autophagy by targeting miR-551b-5p in diabetic cardiomyopathy. Theranostics 9 (15), 4558–4566. 10.7150/thno.31052 31285779 PMC6599651

[B25] Ferro F. Servais S. Besson P. Roger S. Dumas J. F. Brisson L. (2020). Autophagy and mitophagy in cancer metabolic remodelling. Semin. Cell Dev. Biol. 98, 129–138. 10.1016/j.semcdb.2019.05.029 31154012

[B26] Filomeni G. De Zio D. Cecconi F. (2015). Oxidative stress and autophagy: the clash between damage and metabolic needs. Cell Death Differ. 22 (3), 377–388. 10.1038/cdd.2014.150 25257172 PMC4326572

[B27] Forbes J. M. Cooper M. E. (2013). Mechanisms of diabetic complications. Physiol. Rev. 93 (1), 137–188. 10.1152/physrev.00045.2011 23303908

[B28] Frendo-Cumbo S. Tokarz V. L. Bilan P. J. Brumell J. H. Klip A. (2021). Communication between autophagy and insulin action: at the crux of insulin action-insulin resistance? Front. Cell Dev. Biol. l9, 708431. 10.3389/fcell.2021.708431 PMC8319997 34336862

[B29] Fu D. Yu J. Y. Yang S. Wu M. Hammad S. M. Connell A. R. (2016). Survival or death: a dual role for autophagy in stress-induced pericyte loss in diabetic retinopathy. Diabetologia 59 (10), 2251–2261. 10.1007/s00125-016-4058-5 27475954 PMC5016562

[B30] Gong Q. Wang H. Yu P. Qian T. Xu X. (2021). Protective or harmful: the dual roles of autophagy in diabetic retinopathy. Front. Med. 8, 644121. 10.3389/fmed.2021.644121 PMC8026897 33842506

[B31] Guo X. Shi Y. Du P. Wang J. Han Y. Sun B. (2019). HMGB1/TLR4 promotes apoptosis and reduces autophagy of hippocampal neurons in diabetes combined with OSA. Life Sci. 239, 117020. 10.1016/j.lfs.2019.117020 31678553

[B32] Ha J. Choi D. W. Kim K. J. Cho S. Y. Kim H. Kim K. Y. (2021). Association of metformin use with Alzheimer's disease in patients with newly diagnosed type 2 diabetes: a population-based nested case-control study. Sci. Rep. 11 (1), 24069. 10.1038/s41598-021-03406-5 34912022 PMC8674300

[B33] Han Y. Hu Z. Cui A. Liu Z. Ma F. Xue Y. (2019). Post-translational regulation of lipogenesis via AMPK-dependent phosphorylation of insulin-induced gene. Nat. Commun. 10 (1), 623. 10.1038/s41467-019-08585-4 30733434 PMC6367348

[B34] Hara T. Takamura A. Kishi C. Iemura S. Natsume T. Guan J. L. (2008). FIP200, a ULK-interacting protein, is required for autophagosome formation in mammalian cells. J. Cell Biol. 181 (3), 497–510. 10.1083/jcb.200712064 18443221 PMC2364687

[B35] Herzig S. Shaw R. J. (2018). Ampk: guardian of metabolism and mitochondrial homeostasis. Nat. Rev. Mol. Cell Biol. 19 (2), 121–135. 10.1038/nrm.2017.95 28974774 PMC5780224

[B36] Jin J. Shi Y. Gong J. Zhao L. Li Y. He Q. (2019). Exosome secreted from adipose-derived stem cells attenuates diabetic nephropathy by promoting autophagy flux and inhibiting apoptosis in podocyte. Stem Cell Res. Ther. 10 (1), 95. 10.1186/s13287-019-1177-1 30876481 PMC6419838

[B37] Kallergi E. Nikoletopoulou V. (2021). Macroautophagy and normal aging of the nervous system: lessons from animal models. Cell Stress 5 (10), 146–166. 10.15698/cst2021.10.257 34708187 PMC8490955

[B38] Kanamori H. Naruse G. Yoshida A. Minatoguchi S. Watanabe T. Kawaguchi T. (2021). Morphological characteristics in diabetic cardiomyopathy associated with autophagy. J. Cardiol. 77 (1), 30–40. 10.1016/j.jjcc.2020.05.009 32907780

[B39] Kaushik S. Cuervo A. M. (2018). The coming of age of chaperone-mediated autophagy. Nat. Rev. Mol. Cell Biol. 19 (6), 365–381. 10.1038/s41580-018-0001-6 29626215 PMC6399518

[B40] Kelly J. Karlsen M. Steinke G. (2020). Type 2 diabetes remission and lifestyle medicine: a position statement from the American college of lifestyle medicine. Am. J. Lifestyle Med. 14 (4), 406–419. 10.1177/1559827620930962 33281521 PMC7692017

[B41] Kezic A. Popovic L. Lalic K. (2018). mTOR inhibitor therapy and metabolic consequences: where do we stand? Oxid. Med. Cell. Longev. 2018, 2640342. 10.1155/2018/2640342 30034573 PMC6035806

[B42] Kim J. Guan K. L. (2013). AMPK connects energy stress to PIK3C3/VPS34 regulation. Autophagy 9 (7), 1110–1111. 10.4161/auto.24877 23669030 PMC3722323

[B43] Kim J. Kim Y. C. Fang C. Russell R. C. Kim J. H. Fan W. (2013). Differential regulation of distinct Vps34 complexes by AMPK in nutrient stress and autophagy. Cell 152 (1-2), 290–303. 10.1016/j.cell.2012.12.016 23332761 PMC3587159

[B44] Kim J. Kundu M. Viollet B. Guan K. L. (2011). AMPK and mTOR regulate autophagy through direct phosphorylation of Ulk1. Nat. Cell Biol. 13 (2), 132–141. 10.1038/ncb2152 21258367 PMC3987946

[B45] Kim K. H. Lee M. S. (2014). Autophagy--a key player in cellular and body metabolism. Nat. Rev. Endocrinol. 10 (6), 322–337. 10.1038/nrendo.2014.35 24663220

[B46] Kim Y. C. Guan K. L. (2015). mTOR: a pharmacologic target for autophagy regulation. J. Clin. Invest. 125 (1), 25–32. 10.1172/jci73939 25654547 PMC4382265

[B47] Kong F. J. Ma L. L. Guo J. J. Xu L. H. Li Y. Qu S. (2018). Endoplasmic reticulum stress/autophagy pathway is involved in diabetes-induced neuronal apoptosis and cognitive decline in mice. Clin. Sci. 132 (1), 111–125. 10.1042/cs20171432 29212786

[B48] Korbut A. I. Taskaeva I. S. Bgatova N. P. Muraleva N. A. Orlov N. B. Dashkin M. V. (2020). SGLT2 inhibitor empagliflozin and DPP4 inhibitor linagliptin reactivate glomerular autophagy in db/db mice, a model of type 2 diabetes. Int. J. Mol. Sci. 21 (8), E2987. 10.3390/ijms21082987 32340263 PMC7215949

[B49] Krause G. J. Cuervo A. M. (2021). Assessment of mammalian endosomal microautophagy. Methods Cell Biol. 164, 167–185. 10.1016/bs.mcb.2020.10.009 34225914 PMC8826487

[B50] Kubli D. A. Gustafsson Å. B. (2015). Unbreak my heart: targeting mitochondrial autophagy in diabetic cardiomyopathy. Antioxid. Redox Signal. 22 (17), 1527–1544. 10.1089/ars.2015.6322 25808102 PMC4449713

[B51] Lavallard V. J. Meijer A. J. Codogno P. Gual P. (2012). Autophagy, signaling and obesity. Pharmacol. Res. 66 (6), 513–525. 10.1016/j.phrs.2012.09.003 22982482

[B52] Lenoir O. Jasiek M. Hénique C. Guyonnet L. Hartleben B. Bork T. (2015). Endothelial cell and podocyte autophagy synergistically protect from diabetes-induced glomerulosclerosis. Autophagy 11 (7), 1130–1145. 10.1080/15548627.2015.1049799 26039325 PMC4590611

[B53] Levine B. Kroemer G. (2019). Biological functions of autophagy genes: a disease perspective. Cell 176 (1-2), 11–42. 10.1016/j.cell.2018.09.048 30633901 PMC6347410

[B54] Li L. Tan J. Miao Y. Lei P. Zhang Q. (2015). ROS and autophagy: interactions and molecular regulatory mechanisms. Cell. Mol. Neurobiol. 35 (5), 615–621. 10.1007/s10571-015-0166-x 25722131 PMC11486209

[B55] Li W. W. Li J. Bao J. K. (2012). Microautophagy: lesser-known self-eating. Cell. Mol. Life Sci. 69 (7), 1125–1136. 10.1007/s00018-011-0865-5 22080117 PMC11114512

[B56] Lim H. Lim Y. M. Kim K. H. Jeon Y. E. Park K. Kim J. (2018). A novel autophagy enhancer as a therapeutic agent against metabolic syndrome and diabetes. Nat. Commun. 9 (1), 1438. 10.1038/s41467-018-03939-w 29650965 PMC5897400

[B57] Lim S. W. Jin L. Jin J. Yang C. W. (2016). Effect of exendin-4 on autophagy clearance in beta cell of rats with tacrolimus-induced diabetes mellitus. Sci. Rep. 6, 29921. 10.1038/srep29921 27436514 PMC4951772

[B58] Lin K. Y. Hsih W. H. Lin Y. B. Wen C. Y. Chang T. J. (2021). Update in the epidemiology, risk factors, screening, and treatment of diabetic retinopathy. J. Diabetes Investig. 12 (8), 1322–1325. 10.1111/jdi.13480 PMC8354492 33316144

[B59] Liu B. Y. Li L. Bai L. W. Xu C. S. (2021). Long non-coding RNA XIST attenuates diabetic peripheral neuropathy by inducing autophagy through MicroRNA-30d-5p/sirtuin1 Axis. Front. Mol. Biosci. 8, 655157. 10.3389/fmolb.2021.655157 33996907 PMC8113765

[B60] Liu W. J. Huang W. F. Ye L. Chen R. H. Yang C. Wu H. L. (2018). The activity and role of autophagy in the pathogenesis of diabetic nephropathy. Eur. Rev. Med. Pharmacol. Sci. 22 (10), 3182–3189. 10.26355/eurrev_201805_15079 29863264

[B61] Liu X. Q. Jiang L. Li Y. Y. Huang Y. B. Hu X. R. Zhu W. (2022). Wogonin protects glomerular podocytes by targeting Bcl-2-mediated autophagy and apoptosis in diabetic kidney disease. Acta Pharmacol. Sin. 43 (1), 96–110. 10.1038/s41401-021-00721-5 34253875 PMC8724322

[B62] Lopes de Faria J. M. Duarte D. A. Montemurro C. Papadimitriou A. Consonni S. R. Lopes de Faria J. B. (2016). Defective autophagy in diabetic retinopathy. Invest. Ophthalmol. Vis. Sci. 57 (10), 4356–4366. 10.1167/iovs.16-19197 27564518

[B63] Ma X. Liu Z. Ilyas I. Little P. J. Kamato D. Sahebka A. (2021). GLP-1 receptor agonists (GLP-1RAs): cardiovascular actions and therapeutic potential. Int. J. Biol. Sci. 17 (8), 2050–2068. 10.7150/ijbs.59965 34131405 PMC8193264

[B64] Madhavi Y. V. Gaikwad N. Yerra V. G. Kalvala A. K. Nanduri S. Kumar A. (2019). Targeting AMPK in diabetes and diabetic complications: energy homeostasis, autophagy and mitochondrial health. Curr. Med. Chem. 26 (27), 5207–5229. 10.2174/0929867325666180406120051 29623826

[B65] Malone J. I. Knox G. Harvey C. (1984). Sorbitol accumulation is altered in type 1 (insulin-dependent) diabetes mellitus. Diabetologia 27 (5), 509–513. 10.1007/BF00290385 6510596

[B66] Marsh S. A. Powell P. C. Dell'italia L. J. Chatham J. C. (2013). Cardiac O-GlcNAcylation blunts autophagic signaling in the diabetic heart. Life Sci. 92 (11), 648–656. 10.1016/j.lfs.2012.06.011 22728715 PMC3477499

[B67] Mizushima N. Levine B. (2020). Autophagy in human diseases. N. Engl. J. Med. 383 (16), 1564–1576. 10.1056/NEJMra2022774 33053285

[B68] Mizushima N. (2010). The role of the Atg1/ULK1 complex in autophagy regulation. Curr. Opin. Cell Biol. 22 (2), 132–139. 10.1016/j.ceb.2009.12.004 20056399

[B69] Mohseni S. (2011). Autophagy in insulin-induced hypoglycaemic neuropathy. Pathology 43 (3), 254–260. 10.1097/PAT.0b013e328343c992 21436636

[B70] Münz C. (2021). The macroautophagy machinery in MHC restricted antigen presentation. Front. Immunol. 12, 628429. 10.3389/fimmu.2021.628429 33717153 PMC7947692

[B71] Muralidharan C. Linnemann A. K. (2021). β-Cell autophagy in the pathogenesis of type 1 diabetes. Am. J. Physiol. Endocrinol. Metab. 321 (3), E410–e416. 10.1152/ajpendo.00151.2021 34338043 PMC8461796

[B72] Muriach M. Flores-Bellver M. Romero F. J. Barcia J. M. (2014). Diabetes and the brain: oxidative stress, inflammation, and autophagy. Oxid. Med. Cell. Longev. 2014, 102158. 10.1155/2014/102158 25215171 PMC4158559

[B73] Naguib M. Tarabay A. ElSaraf N. Rashed L. ElMeligy A. (2021). Beclin1 circulating level as predictor of carotid intima-media thickness in patients with type 2 diabetes mellitus. Med. Baltim. 100 (28), e26630. 10.1097/md.0000000000026630 PMC8284749 34260553

[B74] NCD-RisC (2016). Worldwide trends in diabetes since 1980: a pooled analysis of 751 population-based studies with 4.4 million participants. Lancet 387 (10027), 1513–1530. 10.1016/s0140-6736(16)00618-8 27061677 PMC5081106

[B75] Nguyen T. T. Ta Q. T. H. Nguyen T. K. O. Nguyen T. T. D. Giau V. V. (2020). Type 3 diabetes and its role implications in Alzheimer's disease. Int. J. Mol. Sci. 21 (9), 3165. 10.3390/ijms21093165 PMC7246646 32365816

[B76] Nieto-Torres J. L. Hansen M. (2021). Macroautophagy and aging: the impact of cellular recycling on health and longevity. Mol. Asp. Med. 82, 101020. 10.1016/j.mam.2021.101020 PMC8671213 34507801

[B77] Packer M. (2020). Autophagy-dependent and -independent modulation of oxidative and organellar stress in the diabetic heart by glucose-lowering drugs. Cardiovasc. Diabetol. 19 (1), 62. 10.1186/s12933-020-01041-4 32404204 PMC7222526

[B78] Papadopoulou-Marketou N. Chrousos G. P. Kanaka-Gantenbein C. (2017). Diabetic nephropathy in type 1 diabetes: a review of early natural history, pathogenesis, and diagnosis. Diabetes. Metab. Res. Rev. 33 (2), e2841. 10.1002/dmrr.2841 27457509

[B79] Peng K. Y. Horng L. Y. Sung H. C. Huang H. C. Wu R. T. (2011). Hepatocyte growth factor has a role in the amelioration of diabetic vascular complications via autophagic clearance of advanced glycation end products: dispo85E, an HGF inducer, as a potential botanical drug. Metabolism. 60 (6), 888–892. 10.1016/j.metabol.2010.08.009 21040934

[B80] Pohl C. Dikic I. (2019). Cellular quality control by the ubiquitin-proteasome system and autophagy. Science 366 (6467), 818–822. 10.1126/science.aax3769 31727826

[B81] Qi Z. Chen L. (2019). Endoplasmic reticulum stress and autophagy. Adv. Exp. Med. Biol. 1206, 167–177. 10.1007/978-981-15-0602-4_8 31776985

[B82] Ravanan P. Srikumar I. F. Talwar P. (2017). Autophagy: the spotlight for cellular stress responses. Life Sci. 188, 53–67. 10.1016/j.lfs.2017.08.029 28866100

[B83] Rawal S. Manning P. Katare R. (2014). Cardiovascular microRNAs: as modulators and diagnostic biomarkers of diabetic heart disease. Cardiovasc. Diabetol. 13, 44. 10.1186/1475-2840-13-44 24528626 PMC3976030

[B84] Ritchie R. H. Abel E. D. (2020). Basic mechanisms of diabetic heart disease. Circ. Res. 126 (11), 1501–1525. 10.1161/circresaha.120.315913 32437308 PMC7251974

[B85] Rosa M. D. Distefano G. Gagliano C. Rusciano D. Malaguarnera L. (2016). Autophagy in diabetic retinopathy. Curr. Neuropharmacol. 14 (8), 810–825. 10.2174/1570159x14666160321122900 26997506 PMC5333581

[B86] Sanajou D. Ghorbani Haghjo A. Argani H. Aslani S. (2018). AGE-RAGE axis blockade in diabetic nephropathy: current status and future directions. Eur. J. Pharmacol. 833, 158–164. 10.1016/j.ejphar.2018.06.001 29883668

[B87] Saxton R. A. Sabatini D. M. (2017). mTOR signaling in growth, metabolism, and disease. Cell 168 (6), 960–976. 10.1016/j.cell.2017.02.004 28283069 PMC5394987

[B88] Schuck S. (2020). Microautophagy - distinct molecular mechanisms handle cargoes of many sizes. J. Cell Sci. 133 (17), jcs246322. 10.1242/jcs.246322 32907930

[B89] Selvarajah D. Kar D. Khunti K. Davies M. J. Scott A. R. Walker J. (2019). Diabetic peripheral neuropathy: advances in diagnosis and strategies for screening and early intervention. Lancet. Diabetes Endocrinol. 7 (12), 938–948. 10.1016/s2213-8587(19)30081-6 31624024

[B90] Sermersheim M. A. Park K. H. Gumpper K. Adesanya T. M. Song K. Tan T. (2017). MicroRNA regulation of autophagy in cardiovascular disease. Front. Biosci. 22, 48–65. 10.2741/4471 PMC5349319 27814601

[B91] Sha Y. Rao L. Settembre C. Ballabio A. Eissa N. T. (2017). STUB1 regulates TFEB-induced autophagy-lysosome pathway. Embo J. 36 (17), 2544–2552. 10.15252/embj.201796699 28754656 PMC5579343

[B92] Shin H. J. Kim H. Oh S. Lee J. G. Kee M. Ko H. J. (2016). AMPK-SKP2-CARM1 signalling cascade in transcriptional regulation of autophagy. Nature 534 (7608), 553–557. 10.1038/nature18014 27309807 PMC5568428

[B93] Sieńko K. Poormassalehgoo A. Yamada K. Goto-Yamada S. (2020). Microautophagy in plants: consideration of its molecular mechanism. Cells 9 (4), E887. 10.3390/cells9040887 32260410 PMC7226842

[B94] Soldevila-Domenech N. Cuenca-Royo A. Babio N. Forcano L. Nishi S. Vintró-Alcaraz C. (2021). Metformin use and cognitive function in older adults with type 2 diabetes following a mediterranean diet intervention. Front. Nutr. 8, 742586. 10.3389/fnut.2021.742586 34676236 PMC8523839

[B95] Song S. Tan J. Miao Y. Zhang Q. (2018). Crosstalk of ER stress-mediated autophagy and ER-phagy: involvement of UPR and the core autophagy machinery. J. Cell. Physiol. 233 (5), 3867–3874. 10.1002/jcp.26137 28777470

[B96] Su J. Zhou L. Kong X. Yang X. Xiang X. Zhang Y. (2013). Endoplasmic reticulum is at the crossroads of autophagy, inflammation, and apoptosis signaling pathways and participates in the pathogenesis of diabetes mellitus. J. Diabetes Res. 2013, 193461. 10.1155/2013/193461 23762873 PMC3673337

[B97] Sun J. Lu H. Liang W. Zhao G. Ren L. Hu D. (2021). Endothelial TFEB (transcription factor EB) improves glucose tolerance via upregulation of IRS (insulin receptor substrate) 1 and IRS2. Arterioscler. Thromb. Vasc. Biol. 41 (2), 783–795. 10.1161/atvbaha.120.315310 33297755 PMC8105265

[B98] Sun K. Wang W. Wang C. Lao G. Liu D. Mai L. (2016). AGEs trigger autophagy in diabetic skin tissues and fibroblasts. Biochem. Biophys. Res. Commun. 471 (3), 355–360. 10.1016/j.bbrc.2016.02.020 26872427

[B99] Tagawa A. Yasuda M. Kume S. Yamahara K. Nakazawa J. Chin-Kanasaki M. (2016). Impaired podocyte autophagy exacerbates proteinuria in diabetic nephropathy. Diabetes 65 (3), 755–767. 10.2337/db15-0473 26384385

[B100] Tamargo-Gómez I. Mariño G. (2018). Ampk: regulation of metabolic dynamics in the context of autophagy. Int. J. Mol. Sci. 19 (12), E3812. 10.3390/ijms19123812 30501132 PMC6321489

[B101] Tekirdag K. Cuervo A. M. (2018). Chaperone-mediated autophagy and endosomal microautophagy: joint by a chaperone. J. Biol. Chem. 293 (15), 5414–5424. 10.1074/jbc.R117.818237 29247007 PMC5900761

[B102] Tong M. Saito T. Zhai P. Oka S. I. Mizushima W. Nakamura M. (2019). Mitophagy is essential for maintaining cardiac function during high fat diet-induced diabetic cardiomyopathy. Circ. Res. 124 (9), 1360–1371. 10.1161/circresaha.118.314607 30786833 PMC6483841

[B103] Vergès B. Cariou B. (2015). mTOR inhibitors and diabetes. Diabetes Res. Clin. Pract. 110 (2), 101–108. 10.1016/j.diabres.2015.09.014 26421362

[B104] Wada J. Makino H. (2013). Inflammation and the pathogenesis of diabetic nephropathy. Clin. Sci. 124 (3), 139–152. 10.1042/cs20120198 23075333

[B105] Wang F. Xu C. Reece E. A. Li X. Wu Y. Harman C. (2017). Protein kinase C-alpha suppresses autophagy and induces neural tube defects via miR-129-2 in diabetic pregnancy. Nat. Commun. 8, 15182. 10.1038/ncomms15182 28474670 PMC5424165

[B106] Wu J. Wu J. J. Yang L. J. Wei L. X. Zou D. J. (2013). Rosiglitazone protects against palmitate-induced pancreatic beta-cell death by activation of autophagy via 5'-AMP-activated protein kinase modulation. Endocrine 44 (1), 87–98. 10.1007/s12020-012-9826-5 23109223

[B107] Wu W. H. Zhang M. P. Zhang F. Liu F. Hu Z. X. Hu Q. D. (2011). The role of programmed cell death in streptozotocin-induced early diabetic nephropathy. J. Endocrinol. Invest. 34 (9), e296–301. 10.3275/7741 21597317

[B108] Xiong Y. Zhou L. (2019). The signaling of cellular senescence in diabetic nephropathy. Oxid. Med. Cell. Longev. 2019, 7495629. 10.1155/2019/7495629 31687085 PMC6794967

[B109] Xu H. D. Qin Z. H. (2019). Beclin 1, bcl-2 and autophagy. Adv. Exp. Med. Biol. 1206, 109–126. 10.1007/978-981-15-0602-4_5 31776982

[B110] Xu L. Xu K. Wu Z. Chen Z. He Y. Ma C. (2020). Pioglitazone attenuates advanced glycation end products-induced apoptosis and calcification by modulating autophagy in tendon-derived stem cells. J. Cell. Mol. Med. 24 (3), 2240–2251. 10.1111/jcmm.14901 31957239 PMC7011144

[B111] Xu X. Sun Y. Cen X. Shan B. Zhao Q. Xie T. (2021). Metformin activates chaperone-mediated autophagy and improves disease pathologies in an Alzheimer disease mouse model. Protein Cell 12 (10), 769–787. 10.1007/s13238-021-00858-3 34291435 PMC8464644

[B112] Xue L. Pan Z. Yin Q. Zhang P. Zhang J. Qi W. (2019). Liraglutide promotes autophagy by regulating the AMPK/mTOR pathway in a rat remnant kidney model of chronic renal failure. Int. Urol. Nephrol. 51 (12), 2305–2313. 10.1007/s11255-019-02274-3 31531806

[B113] Yamamoto S. Kuramoto K. Wang N. Situ X. Priyadarshini M. Zhang W. (2018). Autophagy differentially regulates insulin production and insulin sensitivity. Cell Rep. 23 (11), 3286–3299. 10.1016/j.celrep.2018.05.032 29898399 PMC6054876

[B114] Yamamoto Y. H. Noda T. (2020). Autophagosome formation in relation to the endoplasmic reticulum. J. Biomed. Sci. 27 (1), 97. 10.1186/s12929-020-00691-6 33087127 PMC7579975

[B115] Yan M. Wang H. Gu Y. Li X. Tao L. Lu P. (2021). Melatonin exerts protective effects on diabetic retinopathy via inhibition of Wnt/β-catenin pathway as revealed by quantitative proteomics. Exp. Eye Res. 205, 108521. 10.1016/j.exer.2021.108521 33636209

[B116] Yan W. Pang M. Yu Y. Gou X. Si P. Zhawatibai A. (2019). The neuroprotection of liraglutide on diabetic cognitive deficits is associated with improved hippocampal synapses and inhibited neuronal apoptosis. Life Sci. 231, 116566. 10.1016/j.lfs.2019.116566 31201846

[B117] Yang C. Chen X. C. Li Z. H. Wu H. L. Jing K. P. Huang X. R. (2021). SMAD3 promotes autophagy dysregulation by triggering lysosome depletion in tubular epithelial cells in diabetic nephropathy. Autophagy 17 (9), 2325–2344. 10.1080/15548627.2020.1824694 33043774 PMC8496726

[B118] Yang D D. Livingston M. J. Liu Z. Dong G. Zhang M. Chen J. K. (2018). Autophagy in diabetic kidney disease: regulation, pathological role and therapeutic potential. Cell. Mol. Life Sci. 75 (4), 669–688. 10.1007/s00018-017-2639-1 28871310 PMC5771948

[B119] Yang H H. Feng A. Lin S. Yu L. Lin X. Yan X. (2018). Fibroblast growth factor-21 prevents diabetic cardiomyopathy via AMPK-mediated antioxidation and lipid-lowering effects in the heart. Cell Death Dis. 9 (2), 227. 10.1038/s41419-018-0307-5 29445083 PMC5833682

[B120] Yang J. Zhou R. Ma Z. (2019). Autophagy and energy metabolism. Adv. Exp. Med. Biol. 1206, 329–357. 10.1007/978-981-15-0602-4_16 31776993

[B121] Yao J. Tao Z. F. Li C. P. Li X. M. Cao G. F. Jiang Q. (2014). Regulation of autophagy by high glucose in human retinal pigment epithelium. Cell. Physiol. biochem. 33 (1), 107–116. 10.1159/000356654 24481000

[B122] Yao R. Q. Ren C. Xia Z. F. Yao Y. M. (2021). Organelle-specific autophagy in inflammatory diseases: a potential therapeutic target underlying the quality control of multiple organelles. Autophagy 17 (2), 385–401. 10.1080/15548627.2020.1725377 32048886 PMC8007140

[B123] Yassin R. Tadmor H. Farber E. Igbariye A. Armaly-Nakhoul A. Dahan I. (2021). Alteration of autophagy-related protein 5 (ATG5) levels and Atg5 gene expression in diabetes mellitus with and without complications. Diab. Vasc. Dis. Res. 18 (6), 14791641211062050. 10.1177/14791641211062050 34903064 PMC8679033

[B124] Yoo S. M. Jung Y. K. (2018). A molecular approach to mitophagy and mitochondrial dynamics. Mol. Cells 41 (1), 18–26. 10.14348/molcells.2018.2277 29370689 PMC5792708

[B125] Yu S. Y. Dong B. Fang Z. F. Hu X. Q. Tang L. Zhou S. H. (2018). Knockdown of lncRNA AK139328 alleviates myocardial ischaemia/reperfusion injury in diabetic mice via modulating miR-204-3p and inhibiting autophagy. J. Cell. Mol. Med. 22 (10), 4886–4898. 10.1111/jcmm.13754 30047214 PMC6156366

[B126] Yuan Y. Li L. Zhu L. Liu F. Tang X. Liao G. (2020). Mesenchymal stem cells elicit macrophages into M2 phenotype via improving transcription factor EB-mediated autophagy to alleviate diabetic nephropathy. Stem Cells 38 (5), 639–652. 10.1002/stem.3144 31904160

[B127] Yun H. R. Jo Y. H. Kim J. Shin Y. Kim S. S. Choi T. G. (2020). Roles of autophagy in oxidative stress. Int. J. Mol. Sci. 21 (9), E3289. 10.3390/ijms21093289 32384691 PMC7246723

[B128] Zhang D D. Ma Y. Liu J. Deng Y. Zhou B. Wen Y. (2021). Metformin alleviates hepatic steatosis and insulin resistance in a mouse model of high-fat diet-induced nonalcoholic fatty liver disease by promoting transcription factor EB-dependent autophagy. Front. Pharmacol. 12, 689111. 10.3389/fphar.2021.689111 34366846 PMC8346235

[B129] Zhang X X. Zhao S. Yuan Q. Zhu L. Li F. Wang H. (2021). TXNIP, a novel key factor to cause Schwann cell dysfunction in diabetic peripheral neuropathy, under the regulation of PI3K/Akt pathway inhibition-induced DNMT1 and DNMT3a overexpression. Cell Death Dis. 12 (7), 642. 10.1038/s41419-021-03930-2 34162834 PMC8222353

[B130] Zhang X. W. Zhou J. C. Hu Z. W. (2017). Autophagy as a target for development of anti-diabetes drugs derived from natural compounds. J. Asian Nat. Prod. Res. 19 (4), 314–319. 10.1080/10286020.2017.1304929 28347174

[B131] Zhao X. Chen Y. Tan X. Zhang L. Zhang H. Li Z. (2018). Advanced glycation end-products suppress autophagic flux in podocytes by activating mammalian target of rapamycin and inhibiting nuclear translocation of transcription factor EB. J. Pathol. 245 (2), 235–248. 10.1002/path.5077 29570219 PMC5969319

[B132] Zhou W. Yao Y. Li J. Wu D. Zhao M. Yan Z. (2019). TIGAR attenuates high glucose-induced neuronal apoptosis via an autophagy pathway. Front. Mol. Neurosci. 12, 193. 10.3389/fnmol.2019.00193 31456661 PMC6700368

[B133] Zuckerbraun B. (2018). Effects of metformin in a non-diabetic patient population. NCT03772964. Pittsburgh: University of Pittsburgh.

[B134] Zummo F. P. Cullen K. S. Honkanen-Scott M. Shaw J. A. M. Lovat P. E. Arden C. (2017). Glucagon-like peptide 1 protects pancreatic β-cells from death by increasing autophagic flux and restoring lysosomal function. Diabetes 66 (5), 1272–1285. 10.2337/db16-1009 28232493

